# Effects of aerobic and resistance exercise on patients with hypertension: a systematic review and meta-analysis focusing on the sympathetic nervous system

**DOI:** 10.3389/fcvm.2025.1569638

**Published:** 2025-08-18

**Authors:** Jing-feng Wang, Su-jie Mao, Fan Xia, Xiao-lin Li

**Affiliations:** Harbin Sport University Graduate School, Harbin, Heilongjiang, China

**Keywords:** aerobic exercise, resistance exercise, hypertension, autonomic nervous system, meta-analysis

## Abstract

**Background:**

Treatment and control of hypertension are important for the prevention of cardiovascular diseases. The autonomic nervous system plays a major role in the development and progression of hypertension and has become a new research hotspot in cardiovascular disease. Exercise as a non-pharmacologic intervention has likewise received much attention in the field of cardiovascular disease.

**Objective:**

To determine the effects of exercise on the sympathetic and parasympathetic nervous systems of hypertensive patients. The effects of aerobic, resistance, and combined aerobic and resistance exercise on autonomic function in hypertensive patients will be compared and analyzed to explore more appropriate exercise modalities for hypertensive patients.

**Methods:**

Databases such as Web of Science, PubMed, Embase, Cochrane Library, and CNKI were searched to collect randomized controlled trials (RCTs) evaluating exercise (aerobic, resistance, and aerobic combined with resistance exercise) as an intervention for the autonomic nervous system in hypertension. The Cochrane evaluation tool and Jadad scale were used to evaluate the methodological quality of the included literature. RevMan software was used for statistical and sensitivity analyses, and Stata software was used for net analysis and assessment of publication bias.

**Results:**

This study included 20 studies with 794 hypertensive patients. Exercise improved the joint effect sizes of the basic phenotype in hypertensive patients [SMD = 0.89, 95% CI (0.69, 1.10)] as well as blood pressure variability in hypertensive patients [WMD = 0.89, 95% CI (0.51, 1.27)]. The effect of exercise on hypertensive patients was more centered on the sympathetic nervous system [SMD = 0.29, 95% CI (0.17, 0.40)] and was not significant on the parasympathetic nervous system in hypertensive patients [SMD = −0.08, 95% CI (−0.31, 0.14)]. In addition, the efficacy of aerobic combined resistance exercise on the regulation of blood pressure and the autonomic nervous system in hypertensive patients was the most significant (*p* < 0.05).

**Conclusion:**

The regulation of exercise in hypertensive patients is dominated by the sympathetic nervous system. The efficacy of aerobic combined resistance exercise on the autonomic nervous system of hypertensive patients is particularly prominent and plays an important role in improving the blood pressure level of patients, among other things.

**Systematic Review Registration:**

https://www.crd.york.ac.uk/PROSPERO/, identifier CRD42025634362.

## 1 Introduction

Hypertension is a chronic multifactorial disease characterized by a persistent increase in blood pressure (1). As the leading modifiable risk factor for cardiovascular disease morbidity and mortality, with an impact on tens of millions of people worldwide (2). Therefore, active treatment and control of hypertension is important for the prevention of cardiovascular disease (3).

Autonomic nervous system (ANS) dysfunction is a key factor in the development of hypertension (4). The sympathetic and parasympathetic branches of the ANS work in tandem, and the delicate balance between them is critical for cardiovascular stability, effectively regulating key physiological processes such as heart rate and blood pressure (5). In addition, exercise is recognized as an effective means of lowering blood pressure in hypertensive patients and avoiding elevated blood pressure in pre-hypertension (6). For example, in recent years meta-analyses have found that exercise has a significant improvement in the regulation of the autonomic nervous system in hypertensive patients (7, 8). However, despite the numerous studies focusing on exercise to improve autonomic nervous system regulation in hypertensive patients, most of them are limited to overall improvements in indices, and there are no clear conclusions as to whether these improvements predominantly affect the sympathetic nervous system (SNS) or the parasympathetic nervous system (PNS).

Exercise is a key nonpharmacologic intervention for hypertension treatment in all hypertension guidelines (2). Exercise can be broadly categorized into two types, one being aerobic and the other being resistance. Most physical activities are some combination of two types (9). Exercise can be effective in improving ANS balance (10). For example, during exercise, cardiac tone in the vagus nerve gradually subsides, and sympathetic cardiac and vasodilatory tone gradually increases (11). Studies have demonstrated the effects of aerobic, resistance, and aerobic combined resistance exercise on autonomic function improvement (12, 13). For example, differences in the effect of exercise on improving autonomic regulation between hypertensive and normotensive patients were explored in a meta-analysis by Ayesha Miraj Abidi et al. (8). The effect of acute resistance exercise on autonomic activity in hypertensive patients was explored in a meta-analysis by Paulo Farinatti et al. (14). However, there are no systematic evaluations or meta-analyses to assess the differences in the effects of the three exercise modalities, aerobic, resistance, and aerobic combined with resistance, on the improvement of autonomic function in hypertensive patients (15).

Although, a large number of randomized controlled trials have explored the effects of exercise (including aerobic, resistance and combined exercise) on the autonomic nervous system in hypertensive patients, there is heterogeneity in the findings. Existing studies are generally at risk of bias in the original study. Thus, this study aimed to explore the focus of exercise in improving the function of the autonomic nervous system (sympathetic and parasympathetic nervous systems) in hypertensive patients through systematic evaluation and meta-analysis, comparatively analyze the differences in efficacy between different forms of exercise, resolve the differences and controversies in existing studies, provide evidence-based medical evidence for complementary clinical treatments, and promote the development of non-pharmacological treatments of hypertension, thereby improving patients’ quality of life and Health.

## 2 Materials and methods

This study used a systematic review and meta-analysis to comprehensively evaluate the improvement effect of exercise in hypertensive patients, focusing primarily on the autonomic nervous system. The Preferred Reporting Items for Systematic Reviews and Meta-Analysis of Individual Participant Data (PRISMA) checklist was followed to ensure rigor and transparency of the study (16). In addition, to improve the verifiability of the study, the study was registered on the Prospero International Prospective Systematic Review Registry Platform under the registration number CRD42025634362.

### 2.1 Inclusion criteria

This study was a systematic evaluation using PICOS principles to assess the efficacy of exercise (aerobic, resistance and aerobic combined resistance) interventions in hypertensive patients. To ensure the inclusiveness of the study, no restriction was imposed on the type of study during the literature search. Moreover, during the literature selection process, we selected only randomized controlled trials and included only those that met the indicators for judging autonomic nervous system outcomes.

Population: All patients who met the diagnostic criteria for hypertension, regardless of symptom presentation. (The diagnosis of hypertension is based on the criteria of the World Health Organization (WHO) and the International Society of Hypertension (ISH). Grade 1 hypertension: systolic blood pressure between 140 and 159 mmHg, diastolic blood pressure between 90 and 99 mmHg; Grade 2 hypertension: systolic blood pressure between 160 and 179 mmHg, diastolic blood pressure between 100 and 109 mmHg; grade 3 hypertension: systolic blood pressure ≥180 mmHg, diastolic blood pressure ≥110 mmHg). Grade 3 hypertension: systolic blood pressure ≥180 mmHg, diastolic blood pressure ≥110 mmHg).

Intervention: Aerobic exercise, resistance exercise, and combined aerobic resistance exercise.

Comparator: Hypertensive patients without exercise intervention or standard treatment.

Outcome: Resting Indicator: Systolic blood pressure (SBP), diastolic blood pressure (DBP), mean blood pressure (MBP), heart rate (HR). Dynamic indicators: heart rate variability [high frequency power (HF); low frequency power (LF), high frequency power to low frequency power ratio (LF/HF), root mean square of successive RR interval differences (RMSSD), the number of successive RR interval differences greater than 50 ms accounted for the total RR Percentage of the number of phases (pNN50), total power (TP), standard deviation of all normal sinus RR intervals (SDNN), and blood pressure variability [24-h systolic blood pressure coefficient of variation (SBP-24 h CV), daytime systolic blood pressure coefficient of variation (SBP-Daytime CV), nighttime systolic blood pressure coefficient of variation (SBP-Nighttime CV), 24-h diastolic blood pressure coefficient of variation (DBP-24 h CV), daytime diastolic blood pressure coefficient of variation (DBP-Daytime CV), and nighttime diastolic blood pressure coefficient of variation (DBP-Nighttime CV)].

Study design: Randomized controlled trial (RCT).

### 2.2 Exclusion criteria

(1) Non-randomized studies and non-controlled studies were excluded. (2) Case studies or case-specific studies were excluded. (3) Studies that combined dietary modification and other lifestyle changes were excluded. (4) Studies with incomplete or unextractable data of outcome indicators were excluded. (5) Review articles were excluded. (6) Animal experiments were excluded. (7) Duplicate publications were excluded. (8) articles in non-Chinese, non-English, and other languages were excluded. (9) Studies with subjects without hypertension were excluded.

### 2.3 Literature search strategy

To ensure the comprehensiveness of the information sources, this study searched Web of Science, PubMed, Embase, Cochrane Library, China Knowledge Network (CNKI), and Wanfang (WANFANG DATA) databases and also reviewed published literature reviews and meta-analyses to find more relevant references and identify and include more potentially relevant studies. Search terms were combined with MESH terms and free terms and set according to the PICOS principle. (Table 1) The search deadline was December 31, 2024, and it aimed to collect randomized controlled trials on the effects of exercise on the autonomic nervous system in hypertensive patients. The search languages were English and Chinese.

**Table 1:** Literature search strategy and search terms.

Retrieval formula	Subject headings	Free word	Chinese search items
#1	Hypertension	Hypertension OR Blood Pressure, High OR Blood Pressures, High OR High Blood Pressure OR High Blood Pressures	高血压
#2	Exercise	Exercise OR Exercises OR Exercise, Physical OR Exercises, Physical OR Physical Exercise OR Physical Exercises OR Physical Activity OR Activities, Physical OR Activity, Physical OR Physical Activities OR Exercise, Aerobic OR Aerobic Exercise OR Aerobic Exercises OR Exercises, Aerobic OR Exercise, Isometric OR Exercises, Isometric OR Isometric Exercises OR Isometric Exercise OR Acute Exercise OR Acute Exercises OR Exercise, Acute OR Exercises, Acute OR Exercise Training OR Exercise Trainings OR Training, Exercise OR Trainings, Exercise	运动
#3	Aerobic exercise	Aerobic exercise OR Exercises OR Exercise, Physical OR Exercises, Physical OR Physical Exercise OR Physical Exercises OR Physical Activity OR Activities, Physical OR Activity, Physical OR Physical Activities OR Exercise, Aerobic OR Aerobic Exercise OR Aerobic Exercises OR Exercises, Aerobic OR Exercise, Isometric OR Exercises, Isometric OR Isometric Exercises OR Isometric Exercise OR Acute Exercise OR Acute Exercises OR Exercise, Acute OR Exercises, Acute OR Exercise Training OR Exercise Trainings OR Training, Exercise OR Trainings, Exercise	有氧运动
#4	Resistance resistance exercise	Resistance resistance exercise OR Training, Resistance OR Strength Training OR Training, Strength OR Weight-Lifting Strengthening Program OR Strengthening Programs, Weight-Lifting OR Strengthening Program, Weight-Lifting OR Weight Lifting Strengthening Program OR Weight-Lifting Strengthening Programs OR Weight-Lifting Exercise Program OR Exercise Programs, Weight-Lifting OR Exercise Program, Weight-Lifting OR Weight Lifting Exercise Program OR Weight-Lifting Exercise Programs OR Weight-Bearing Strengthening Program OR Strengthening Programs, Weight-Bearing OR Strengthening Program, Weight-Bearing OR Weight Bearing Strengthening Program OR Weight-Bearing Strengthening Programs OR Weight-Bearing Exercise Program OR Exercise Programs, Weight-Bearing OR Exercise Program, Weight-Bearing OR Weight Bearing Exercise Program OR Weight-Bearing Exercise Programs	抗阻运动
#5	Autonomic nerves system	autonomic nerves system OR Autonomic Pathway OR Pathway, Autonomic OR Pathways, Autonomic OR Autonomic Nerves OR Autonomic Nerve OR Nerve, Autonomic OR Nerves, Autonomic	自主神经系统
#6	Sympathetic nervous system	sympathetic nervous system OR Nervous Systems, Sympathetic OR Nervous System, Sympathetic OR Sympathetic Nervous Systems OR Systems, Sympathetic Nervous OR System, Sympathetic Nervous	交感神经系统
#7	Parasympathetic Nervous System	Parasympathetic Nervous System OR Nervous System, Parasympathetic OR Nervous Systems, Parasympathetic OR Parasympathetic Nervous Systems OR System, Parasympathetic Nervous OR Systems, Parasympathetic Nervous	副交感神经系统

### 2.4 Study selection

Jingfeng Wang and Xia Fan conducted an initial screening of all retrieved literature and excluded literature unrelated to the study topic based on the title and abstract. After the initial screening, a detailed reading of the literature was conducted to assess whether it met the inclusion criteria. The rescreening process involved a more rigorous assessment, including full-text reading, assessment of the study design, sample characteristics, and specifics and quality of the intervention. In the event of discrepancies in the screening or eligibility assessment process, Xiaolin Li made the final decision to ensure the fairness and accuracy of the selection process.

### 2.5 Data extraction

For literature included after eligibility assessment, data extraction was performed, including first author, year of publication, type of study population, sample size, age, interventions, substance use, and outcome indicators. For literature with incomplete data descriptions or lacking key information, authors were contacted via email to obtain missing data to ensure data completeness and reliability.

In the data extraction stage, to ensure accuracy, Jingfeng Wang and Fan Xia extracted the mean and standard deviation (SD) of all outcome indicators from the included literature, respectively. If the means and SD could not be obtained directly, they were derived from the extracted variables using the appropriate calculation method. The calculation methods were as follows: (1) Interquartile range (IQR), Meanmedian(symmetricaldistributionofdata), SDIQR/1.35(*n* ≤ 25) or SDIQR/1.5(*n* > 25); (2) Estimated from the range, Mean(max+min)/2, SD(max-min)/4 or SD(max-min)/6; (3) Estimated from *t*-test or *F*-test results, that is, $SD\left(Mean\;difference\;t\sqrt{n^{1}+n^{2}}\right)/\sqrt{n^{1}\;\times \;n^{2}}$; (4) From the results of variance analysis, that is, $SD\sqrt{mean\;square\;between\;groups}$; (5) From the regression analysis results, $SD\left(standard\;error\sqrt{n}\right)/regression\;coefficient$; (6) Estimated from the Kaplan–Meier survival curve, the area under the survival curve (AUC), survival function [S(t)], $Mean\overset{\infty }{\underset{0}{\int }}\;S(t)dt$, SD: needed to combine the variance formula of survival time; (7). If the data were presented in bar charts, WebPlotDigitizer was used to convert the graphical data into quantitative data, and Prism was used to calculate the mean and standard deviation of the data (17). The extracted data results were cross-checked. If any inconsistency was found during the data verification process, Xiaolin Li intervened and made the final decision.

### 2.6 Literature quality evaluation

To ensure the quality of the included studies, we used the Cochrane Risk of Bias Assessment tool and the Jadad scale (18). Cochrane's Risk of Bias Assessment tool addressed aspects such as random allocation, allocation concealment, implementation of blinding, data integrity, selective reporting, and other potential sources of bias. Through a quality assessment chart, the degree of compliance with each criterion was indicated using three color labels (low risk, high risk, and unclear) and was described and analyzed in the study results. The Jadad scale is designed for RCTs to quantify the quality of the literature, standardize study evaluation, guide study involvement, and assist in grading evidence by scoring four items: randomization, blinding, withdrawal and exit, and allocation concealment.

### 2.7 Statistical analysis

Data extraction, organization, and graphing of the included studies were performed using WebPlotDigitizer 4.8, Endnote X9, Excel, and GraphPad Prism 8.0.2, and meta-analysis was performed using Review Manager 5.4 and Stata18 software. Weighted mean difference (WMD) was used when the units of the outcome indicator were the same, and standardized mean difference (SMD) was used when the units of the outcome indicator were different or when the means differed significantly, with a significance level of *p* = 0.05, both expressed as 95% CI. Heterogeneity was assessed based on the *Q*-test combined with the *p*-value of *I*². According to the Cochrane Handbook, a fixed-effects model was selected when the heterogeneity test result *I*² ≤ 50%; a random-effects model was selected when the heterogeneity test result *I*² > 50%; and a random-effects model was selected when both *I*² ≤ 50% and *I*² > 50% in the subgroup analysis (19, 20).

### 2.8 Other analyses

In this study, a subgroup analysis was performed to assess the same types of evaluation metrics as subgroup analysis criteria, specifically divided into basic metrics, heart rate variability, and blood pressure variability. By comparing these corresponding metrics, this allowed for gaining a deeper understanding of where the effects of exercise on the autonomic nervous system were more centered.

Through sensitivity analyses, we further explored sources of outcome heterogeneity. In this process, each outcome indicator was included in the analysis and was excluded on a case-by-case basis to assess its impact on the joint effect size. If the excluded effect sizes were not significantly different from the overall effect sizes and the point estimates were within the 95% CI, this indicated good stability of the findings.

In this study, Egger's test was performed using Stata software to assess overall publication bias. Egger's test assesses bias by analyzing the regression relationship between effect sizes and their standard errors and identifies small sample effects. Small sample studies are prone to random error, which often leads to extreme effect size estimates. Identifying this pattern through the Egger's test allows for the exploration of potential assessment bias in the included literature, thereby increasing the transparency and reliability of this meta-analysis.

## 3 Results

### 3.1 Results of literature analysis

#### 3.1.1 Results of the literature search

**Figure 1:**
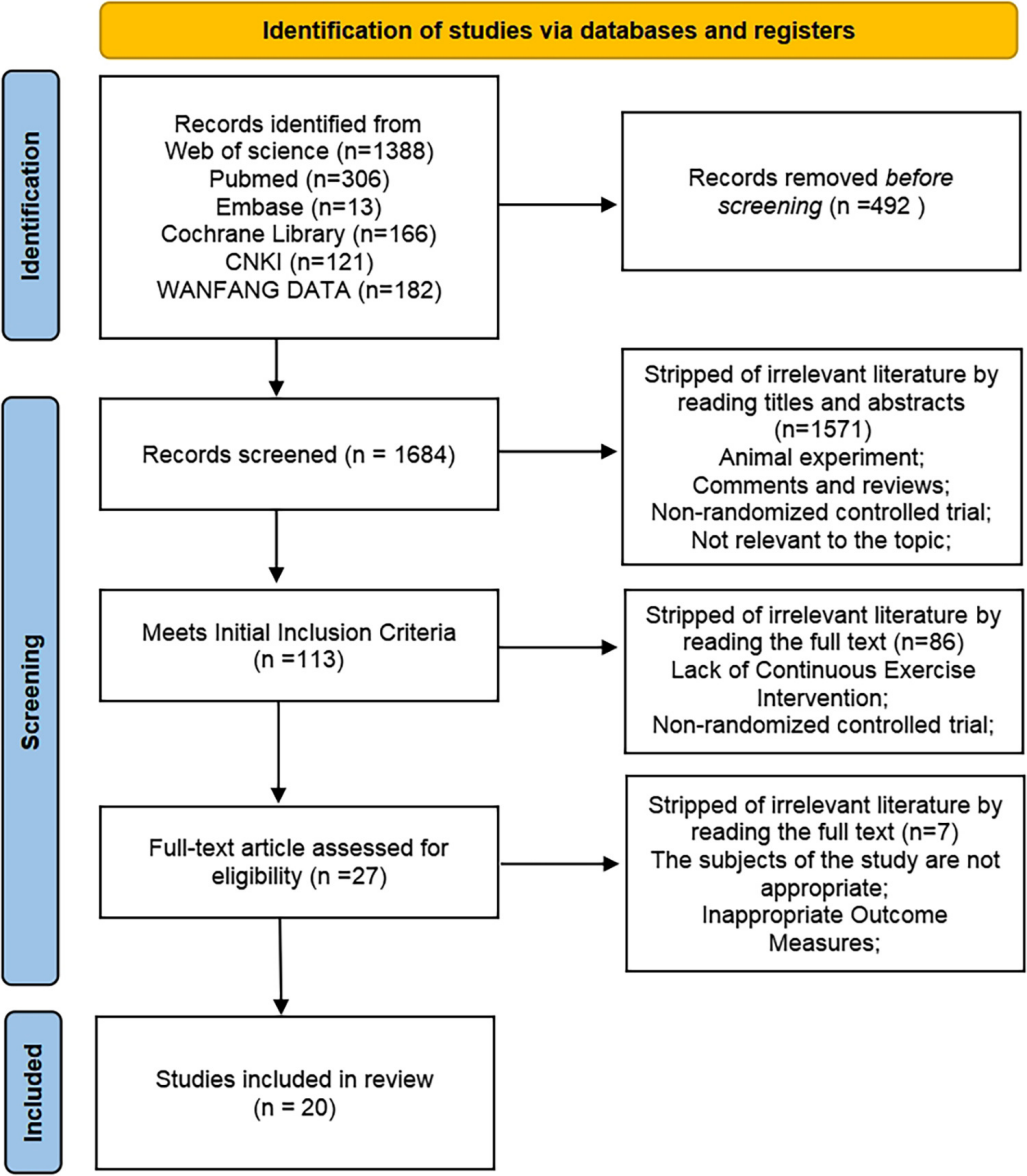
Literature search and screening process.

An initial search for articles related to the effects of exercise on the autonomic nervous system (sympathetic and parasympathetic nervous systems) in hypertensive patients identified a total of 2,176 publications. After exclusion of duplicate articles, 1,684 literatures remained. Based on the inclusion and exclusion criteria, 1,571 articles were further excluded after reading the titles and abstracts, leaving 113 articles for full-text reading. Finally, a total of 20 articles were included in this study (Figure 1).

#### 3.1.2 Characteristics of the included literature

Twenty papers were included in this study for meta-analysis, including 794 participants. There were a total of three exercise modalities studied, including 16 aerobic exercise interventions, four resistance exercise interventions, and four aerobic combined resistance exercise interventions. All controls included in the study did not receive any form of exercise intervention but received usual care, health education, or maintenance of daily activities. A wide range of health-related measurements were included in the literature, such as blood pressure, heart rate, and BMI (Table 2).

**Table 2:** Baseline data before exercise intervention in hypertensive patients.

Researchers; year	Gender	Age mean(SD)	Height (cm)	Body weight(kg)	BMI(kg/㎡)	SBP (mmHg)	DBP (mmHg)	HR (bmp)	Exercise intensity and duration	Mode of exercise	Sample size
Fu et al. (2024) (58)	Male	57.59 (14.2)	165.91 (5.87)	73.92 (13.86)	26.83 (4.77)	130.2 (10)	82.2 (9.3)	75.5 (7.9)	Tai Chi/aerobics: 60 min/time, 40%–60% heart rate reserve, 3 times/week, lasting 12 weeks	AE	43
	Female										37
Rodrigues et al. (2023) (59)	Male	59 (4)	1.62 (0.06)	79.5 (13.1)	29.8 (4.1)	120 (12)	78 (9)	73 (11)	Interval Aerobic exercise: 8 sets of 2-min brisk walking up and down 20 cm steps with 2 min between sets. Continuous aerobic exercise: 30 min of intensity walking at 50% heart rate reserve	AE	12
	Female										
Domingues et al. (2022) (22)	Male	48.4 (8.5)	1.70 (0.1)	86.1 (18)	29.6 (5.2)	127.5 (13.6)	77.2 (11.1)	73 (9.9)	12 min/session, 3 sessions, 2 min interval between sessions	AE	22
	Female										22
Roque Marçal (2022) (2)	Male	68 (7)	N	N	31 (5)	12,618)	74 (9)	75 (9)	High-intensity interval exercise: 21 min of high-intensity aerobic exercise (jogging/running at RPE levels 15–17), alternating with 2 min of active recovery (walking at RPE levels 9–11). Moderate-intensity interval exercise: 26 min of moderate-intensity aerobic exercise (walking at RPE levels 11–13), alternating 2 min of active recovery (walking at RPE levels 9–11). This study adopted a crossover design, meaning that each participant had to undergo all the interventions. As a result, the sample size for each group was the same	AE	9
	Female	67 (7)	N	N	32 (4)	12,618)	74 (9)	76 (9)			11
Sardeli et al. (2022) (60)	Male	65.3 (2.57)	N	78.55 (7.15)	29.66 (2.19)	132.5 (11.21)	82.75 (5.46)	N	Twice a week, on Mondays and Thursdays, strength training (15 min duration) was followed by the aerobic training (50 min duration) and once per week, on Fridays, aerobic training was performed alone (50 min duration), with the protocol consisting of continuous walking and/or running on a treadmill. Strength training consisted of one set of 15 repetitions for each of seven strength exercises for the major muscle groups (leg extension and flexion, leg press, heel lift, bench press, pulley, and abdominal)	CARE	46
	Female										
Fraccari-Pires et al. (2022) (61)	Male	57 (6.68)	N	N	31.5 (3.52)	140.5 (6.7)	81.5 (4.27)	64.5 (5.44)	Aerobic (ARE) session was performed in an electronic treadmill for 45 min at 50%–60% of maximal HR (HRmax) obtained from the ergometric stress test. Resistance(RES) consisted of 6 exercises with 4 sets of 12 submaximal repetitions at moderate intensity (3–5 on the adapted Borg scale). Exercises were performed in the following order: (1) chair squat, (2) vertical bench press, (3) seated knee raise, (4) seated row, (5) dorsiflexion and plantar flexion, and (6) shoulder abduction. A 1 min rest interval was adopted between sets and exercises. All exercises were performed in the full range of motion, and muscle contractions—concentric and eccentric—were performed at moderate velocity (2 s for each). Combined (COM) consisted of AER exercise performed at 50%–60% HRmax for 25 min plus RES based on 6 exercises with 2 sets of 12 submaximal repetitions at moderate intensity according to modified Borg scale. All exercise sessions lasted up to 60 min and were supervised by an exercise physiologist	AE, RE, CARE	9
	Female										11
Caminiti et al. (2021) (50)	Male	68.3 (5.05)	N	87.2 (4.26)	29.75 (1.86)	121.9 (17.28)	80.95 (7.59)	N	Each exercise session included 10 min of warm-up, 10 min of cool-down, and 60 min of aerobic exercise on a treadmill. 40 min of aerobic training on treadmill; 20 min of resistance training consisting in the following exercises: leg press and extension, shoulder press, chest press, low row, and vertical traction. Patients performed 10 repetitions per set, two sets for each exercise at 60% of 1 RM, with 2 min rest between sets	AE, CARE	55
	Female										
Casonatto et al. (2020) (62)	Male	57.15 (10.3)	1.63 (0.06)	78.45 (9.73)	29.35 (3.29)	138.5 (9.22)	84 (5.07)	N	40 min run/walk, 60%–70% of heart rate reserve	AE	20
	Female										
Oliveira-Dantas et al. (2020) (63)	Male	64.7 (4.7)	N	N	28.4 (1.79)	N	N	N	The resistance training intervention period lasted 10 weeks. The supervised group sessions were held twice a week for the first 5 weeks, and after this initial period, the frequency increased to 3 times per week, totaling 25 exercise sessions. The short-term resistance training program consisted of 9 exercises, which were conducted in the following order: seated leg press; seated rowing machine; trunk flexion; knee flexion machine, bench press, trunk extension machine, push press, standing plantar flexion, and front pull-down.	RE	25
	Female										
Brito et al. (2019) (64)	Male	50 (4.66)	1.71 (0.05)	88.45 (7.86)	30.15 (1.88)	133.5 (4.5)	90.5 (3.62)	N	Four weeks before training, the exercise time was increased from 30 min to 45 min. Starting from the fifth week, the intensity was increased every 2 weeks, from the heart rate corresponding to the anaerobic threshold to 10% below the respiratory compensation point	AE	50
	Female										
Masroor et al. (2018) (38)	Male	40.61 (2.47)	154.9 (3.12)	70.7 (5.94)	30.5 (2.54)	145.5 (5.56)	84.8 (3.08)	77.9 (5.51)	Combined aerobic and resistance training trained on an inclined (5.0%) treadmill 3 days/week for 4 weeks. In addition, resistance training was provided twice a week, resulting in a total of 5 training days/week. Each session commenced with a 5-min warm-up performed at 40% of HRmax on treadmill, followed by either an aerobic or resistance exercise bout. Aerobic training was performed at 50%–80% of HRmax (intensity was progressed gradually from 50% to 80% HRmax across 4 weeks) for 20 min, followed by 5 min of cool down at 40% of HRmax. Resistance training constituted 3 sets of 10 repetitions of 5 exercises: bicep curls, triceps extensions, abdominal crunches, leg curls, and knee extensions, at an intensity of 50%–80% of 1RM (intensity was progressed gradually from 50% to 80% of 1 RM over 4 weeks).30, 31 HR, SBP, and DBP were closely monitored during and after exercise. Exercise training was performed 5 times/week for 4 weeks	CARE	28
	Female										
Vale et al. (2018) (65)	Male	57.73 (6.11)	1.56 (0.08)	65.77 (10.37)	26.90 (3.74)	130.5 (8.45)	78.06 (3.65)	67.52 (3.39)	After determination of the loads of 6RM and 15RM, the participants performed three different experimental protocols: a control session, a RT session with 6RM, and a RT session with 15RM. The rest intervals between sets and exercises lasted for 2 min	RE	15
	Female										
Pushpanathan et al. (2015) (66)	Male	43.36 (4.45)	1.64 (0.05)	73.47 (7.39)	27.06 (2.38)	125.6 (5.57)	81.82 (4.16)	72.63 (6.29)	Yoga: 45 min per session, 3 times a week, for a total of 12 weeks	AE	44
	Female										11
Sun and Bai (2015) (67)	Male	≥60	N	76.87 (11.64)	27.35 (4.76)	147.96 (10.62)	97.61 (5.84)	77.9 (5.1)	3 times per week. The exercise intensity in the first week is 30% of VO_2_ max. From then on, the intensity will be increased by 10% of VO_2_ max each week. When the intensity reaches 60% of VO_2_ max, it will remain constant until the end of the experiment. The exercise lasts for a total of 3 months.	AE	44
	Female										34
Zhang (2015) (68)	Male	48.3 (7)	1.66 (0.04)	71.4 (7.6)	26 (3)	143 (16)	95 (10)	77 (6)	Jogging, 40 min per session, 3 times a week, for a total of 24 weeks. During the first 12 weeks, the exercise intensity was 60%–70% of VO_2_ max, and in the last 12 weeks, it increased to 70%–75% of VO_2_ max	AE	12
	Female										18
Pagonas et al. (2014) (21)	Male	67.7 (43–77)	N	N	29.5 (4.5)	133.1 (12.1)	73.8 (6.4)	N	The initial duration of the training was 30 min. in the first week, the training consisted of five 3-min loads; between loads, the patient walked at half speed for 3 min. in the second week, the duration of the exercise was gradually increased to four 5-min loads per day; in the third week, to three 8-min loads per day; in the fourth week, to three 10-min loads per day; and in the fifth week, to two 15-min loads per day. The sixth week and subsequent weeks, the exercise volume was gradually increased to 30, 32, and 36 min per day and was performed without interruption. Weeks, the amount of exercise was gradually increased to 30, 32, and 36 min per day and was performed without interruption. Training was performed three times a week for 8–12 weeks.	AE	66
	Female										
Huang (2014) (69)	Male	54.4 (8)	1.65 (0.03)	77.4 (12.1)	28.6 (4.6)	145 (8)	96 (4)	77 (4)	Intensity of 60% VO_2_ max for the first 6 weeks, increasing to 70% VO_2_ max for 40 min/session for the second 6 weeks	AE, RE	19
	Female										14
Knoepfli-Lenzin C (2010)	Male	38 (5)	N	N	27 (3)	137.6 (3.848)	90.31 (3.389)	74 (8)	Soccer group: 12 weeks of alternating high and low loads for aerobic training; running group: 12 weeks of continuous running exercise at 80% of maximum heart rate	AE	47
	Female										
Wang et al. (2007) (70)	Male	63.6 (2.5)	155.6 (6.3)	65.7 (7.7	N	140∼160	90∼95	N	Walking: 3 times per week, 1 h each time, exercise intensity 46%–60% heart rate reserve for 12 weeks.	AE	50
	Female										
Laterza et al. (2007) (32)	Male	44 (1)	1.70 (0.02)	77 (3)	26 (1)	145 (4)	94 (2)	76 (3.32)	60 min of exercise three times a week for 4 months. Exercise intensity was determined by the HR level, which corresponded to the anaerobic threshold to 70% VO_2_ max	AE	13
	Female										7

N: No information.

#### 3.1.3 Quality assessment of the included literature

**Figure 2:**
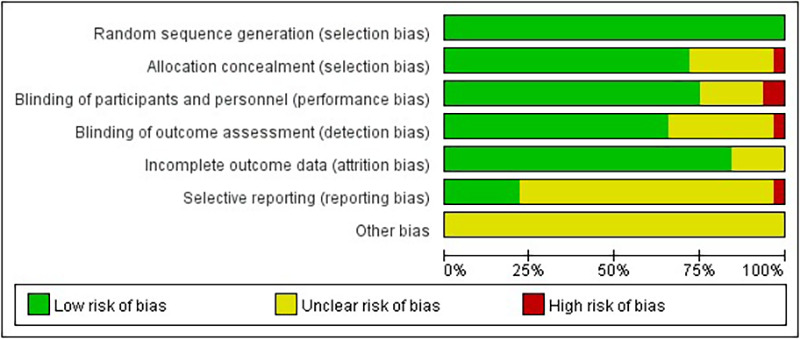
Quality evaluation distribution chart.

In the area of random sequence generation, 20 studies were considered low risk. In the area of allocation concealment, 16 studies also showed low risk. However, four studies did not provide sufficient information on concealment of allocations. In the areas of blinding of participants and personnel and blinding of outcome assessment, 13 and 10 studies implemented rigorous blinding measures and were considered low risk, but 2 studies had significant blinding problems in outcome assessment. In the incomplete outcome data domain, four studies lacked a description of exit. Sensitivity analyses were conducted on these studies to fully understand and explain the impact of missing data on the findings. In the selective reporting domain, none of the 20 studies mentioned this risk. In assessing other potential biases, a systematic evaluation was conducted, and it was concluded that the included studies did not exhibit significant other biases (Figure 2).

A modified version of the Jadad score was assessed for the 20 randomized controlled trials included. All studies used randomized allocation, of which 15 used blinding and the rest were not fully described or were not blinded, reducing the score. 16 trials reported complete withdrawal and dropout, and 4 trials were unclear about complete reporting of withdrawal and dropout. 6 study allocation concealment was not described (Table 3).

**Table 3:** Jadad quality evaluation of randomized controlled trials.

Item	Randomization	Blinding	Withdrawal	Allocation concealment	Totals
Fu et al. (2024) (58)	2	2	2	1	7
Rodrigues et al. (2023) (59)	2	1	2	1	6
Domingues et al. (2022) (22)	2	2	2	0	6
Roque Marçal et al. (2022) (2)	2	1	2	1	6
Sardeli et al. (2022) (60)	2	2	2	1	7
Fraccari-Pires (2022) (61)	2	2	1	1	6
Caminiti et al. (2021) (50)	2	2	2	0	6
Casonatto et al. (2020) (62)	2	2	2	1	7
Oliveira-Dantas et al. (2020) (63)	2	1	2	1	6
Brito et al. (2019) (64)	2	2	2	1	7
Masroor et al. (2018) (38)	2	2	2	0	6
Vale et al. (2018) (65)	2	2	1	1	6
Pushpanathan et al. (2015) (66)	2	2	2	1	7
Sun and Bai (2015) (67)	2	2	1	1	6
Zhang (2015) (68)	2	0	2	1	5
Pagonas et al. (2014) (21)	2	2	2	0	6
Huang (2014) (69)	2	2	2	0	6
Knoepfli-Lenzin (2010) (71)	2	2	2	0	6
Wang et al. (2007) (70)	2	0	2	1	5
Laterza et al. (2007) (32)	2	2	1	1	6

#### 3.1.4 Publication bias analysis

For the basic indicators, there was no publication bias in the SBP, DBP, and HR groups (*p* > 0.05), while there was a publication bias in the MBP group (*p* < 0.05). For the evaluation of the sympathetic nervous system as well as the autonomic nervous system balance, there was no publication bias in the LF (ms²) group, LF (nu), and LF/HF group (*p* > 0.05). For the indexes evaluating the parasympathetic nervous system, there was no publication bias in the HF (ms²), HF (nu), RMSSD, and pNN50% groups (*p* > 0.05). For blood pressure variability indices, publication bias was observed in the SBP-24 h CV group and the DBP-24 h CV group (*p* < 0.05) (Table 4).

**Table 4:** Egger test for literature publication bias results.

Item	Relevant indicators	Egger's test	*p* value
Basic data	SBP	0.454	*p* > 0.05
	DBP	0.311	*p* > 0.05
	MBP	0.017	*p* < 0.05
	HR	0.290	*p* > 0.05
Blood pressure variability	SBP-24 h CV	0.021	*p* < 0.05
	DBP-24-h CV	0.011	*p* < 0.05
Sympathetic nervous system and autonomic nervous system balance	LF (ms²)	0.261	*p* > 0.05
	LF (nu)	0.174	*p* > 0.05
	LF/HF	0.079	*p* > 0.05
Parasympathetic nervous system response	HF (ms²)	0.305	*p* > 0.05
	HF (nu)	0.658	*p* > 0.05
	RMMSD	0.072	*p* > 0.05
	pNN50	0.149	*p* > 0.05
Total effect of autonomic nervous system	TP	0.219	*p* > 0.05
	SDNN	0.301	*p* > 0.05

The data of blood pressure variability indicators (SBP-daytime CV, SBP-nighttime CV, DBP-daytime CV, DBP-nighttime CV) in the included literature were limited, so the publication bias analysis was not conducted.

#### 3.1.5 Sensitivity analysis

Excluding any single study, there was no statistically significant difference between the joint effect size and the overall effect size for the remaining studies. The point estimate after each exclusion consistently fell within the 95% CI of the overall effect size.

#### 3.1.6 Heterogeneity analysis

**Figure 3:**
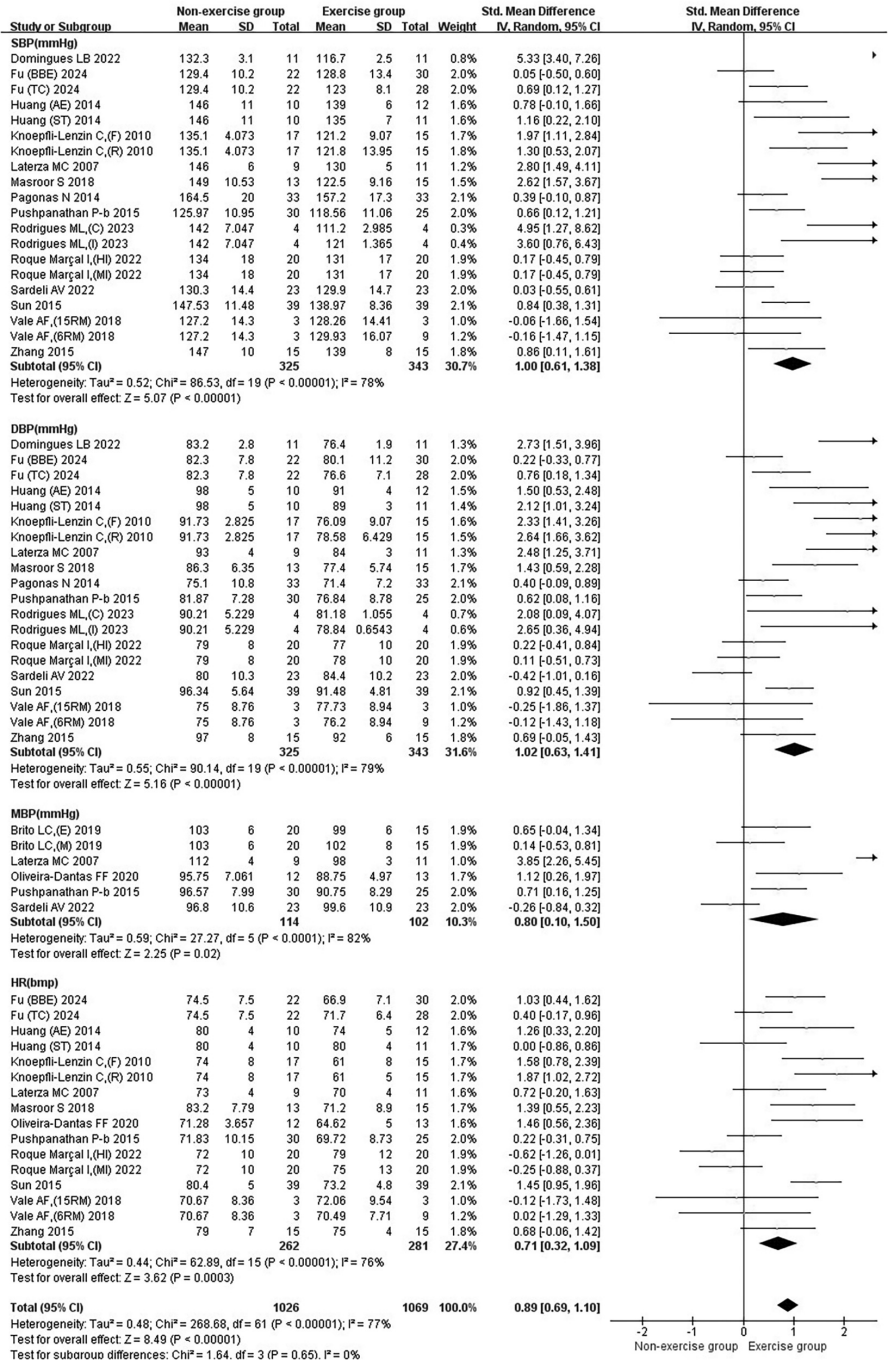
Subgroup analysis of basic indicators in patients with hypertension treated with exercise.

**Figure 4:**
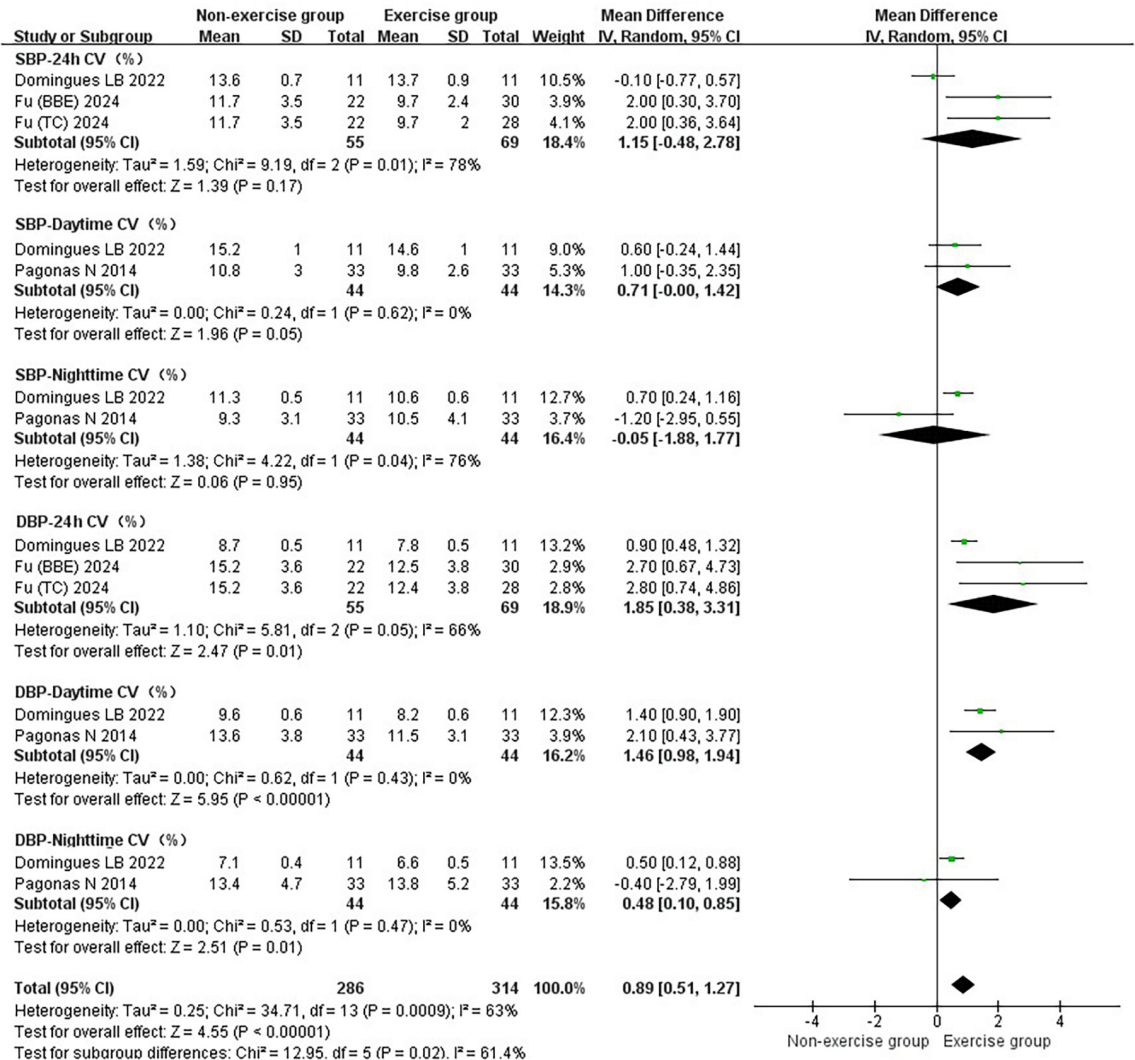
Subgroup analysis of exercise intervention on blood pressure variability in patients with hypertension.

**Figure 5:**
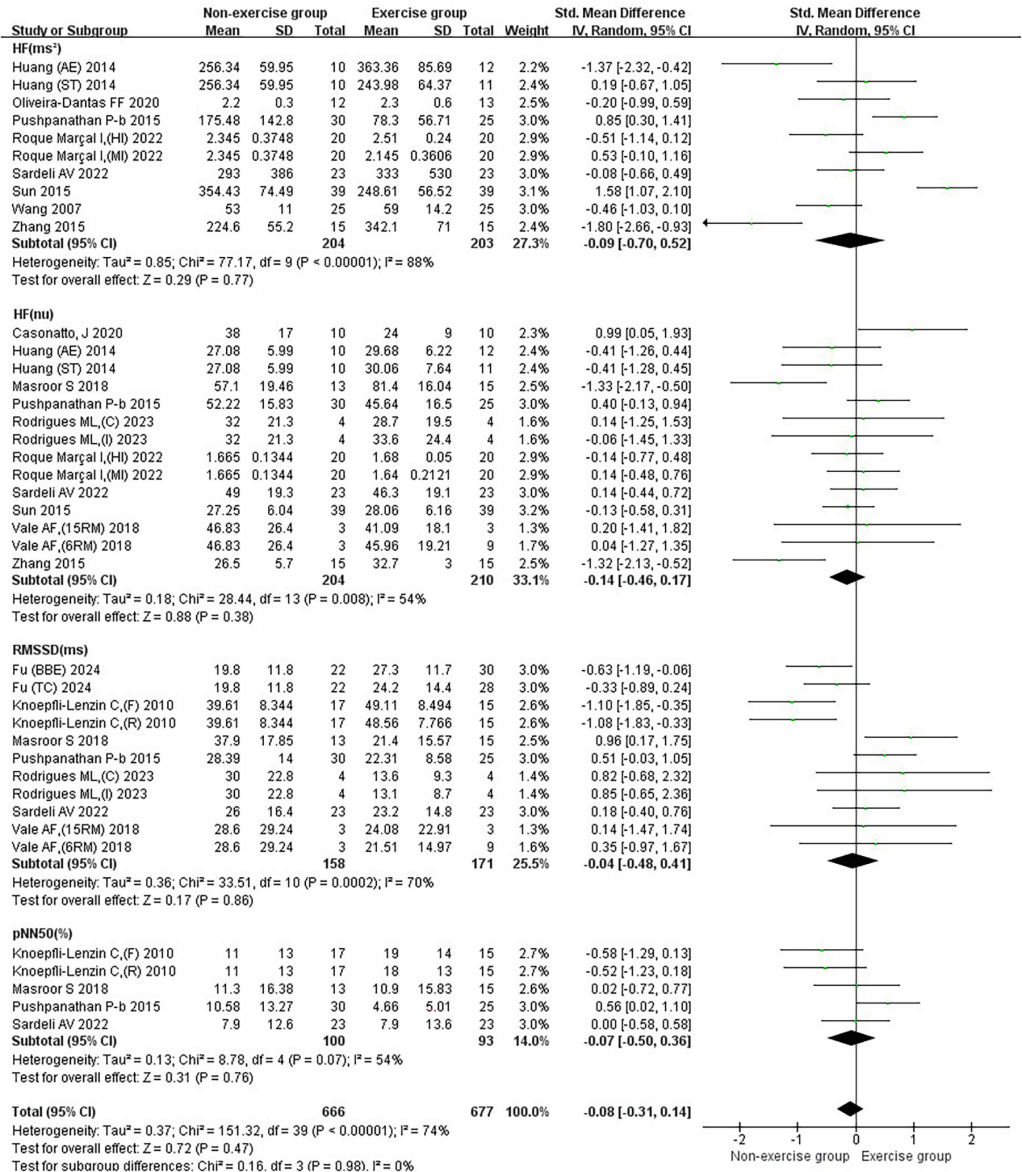
Subgroup analysis of exercise intervention on parasympathetic nervous system in patients with hypertension.

**Figure 6:**
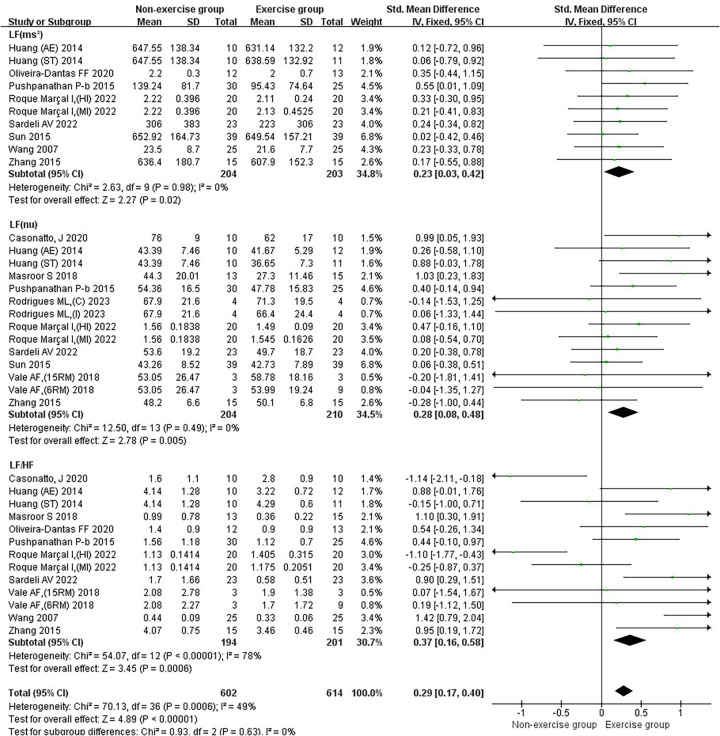
Subgroup analysis of exercise intervention on sympathetic nervous system and autonomic nervous system balance in patients with hypertension.

**Figure 7:**
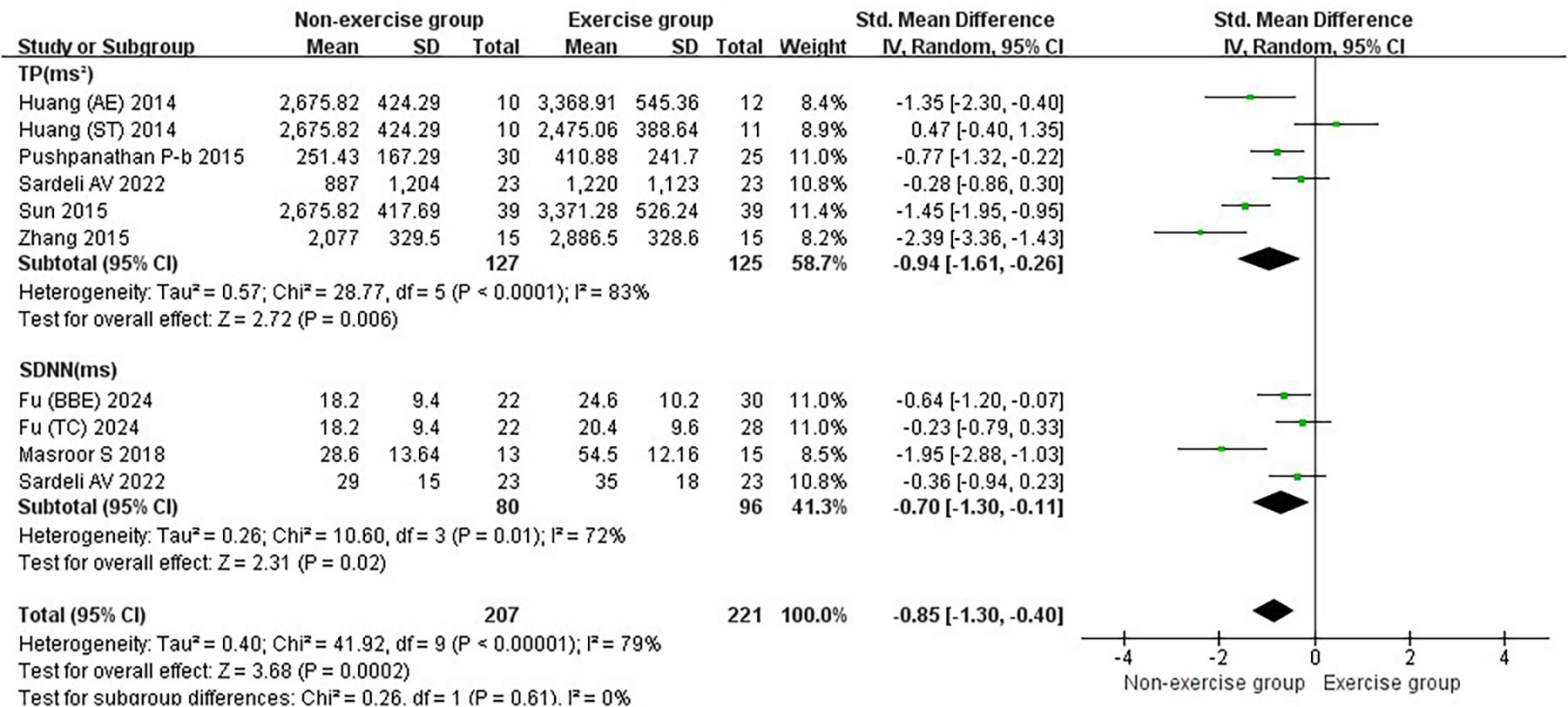
Subgroup analysis of the total effect of exercise intervention on heart rate variability in the frequency domain and time domain in hypertensive patients.

In the subgroup analysis of basic indicators of hypertension patients, the heterogeneity index (*I*²) of SBP was 78%, showing high heterogeneity. The *I*² of DBP was 79%, showing high heterogeneity. The *I*² of MBP was 82%, indicating high heterogeneity. The *I*² of HR was 76%, showing high heterogeneity. The combined effect *I*² was 77%, with high heterogeneity (Figure 3). In the subgroup analysis of blood pressure variability indicators in hypertensive patients, the *I*² of SBP-24 h CV was 78%, showing high heterogeneity. The *I*² of SBP-Daytime CV was 0%, showing low heterogeneity. The *I*² of SBP-Nighttime CV was 76%, showing high heterogeneity. The *I*² of DBP-24 h CV was 66%, showing moderate heterogeneity. The *I*² of DBP-Daytime CV was 0%, indicating low heterogeneity. The *I*² of DBP-Nighttime CV was 0%, with low heterogeneity. The combined effect *I*² was 63%, with moderate heterogeneity (Figure 4). In the subgroup analysis of parasympathetic nervous system indicators in hypertensive patients, the *I*² of HF (ms²) was 88%, showing high heterogeneity. The *I*² of HF (nu) was 54%, showing moderate heterogeneity. The *I*² of RMSSD was 70%, showing moderate heterogeneity. The *I*² of pNN50 was 54%, indicating moderate heterogeneity. The combined effect *I*² was 74%, with moderate heterogeneity (Figure 5). In the subgroup analysis of sympathetic nervous system and autonomic nervous system balance indicators in hypertensive patients, the *I*² of LF(ms²) was 0%, showing low heterogeneity. The *I*² of LF (nu) was 0%, indicating low heterogeneity. The *I*² of LF/HF was 78%, showing moderate heterogeneity. The combined effect *I*² was 49%, with low heterogeneity (Figure 6). In the subgroup analysis of the total frequency domain and time domain indicators of heart rate variability in hypertensive patients, the *I*² of TP was 83%, showing moderate heterogeneity. The *I*² of SDNN was 72%, indicating moderate heterogeneity. The combined effect *I*² was 79%, with high heterogeneity (Figure 7).

### 3.2 Meta-analysis results

#### 3.2.1 Results of the effect of exercise on basic indicators in hypertensive patients

Exercise significantly reduced the joint effect sizes of blood pressure as well as heart rate in hypertensive patients [SMD = 0.89, 95% CI (0.69, 1.10)]. Exercise significantly reduced SBP [SMD = 1.00, 95% CI (0.61, 1.38)], DBP [SMD = 1.02, 95% CI (0.63, 1.41)], MBP [SMD = 0.80, 95% CI (0.10, 1.50)], and HR [SMD = 0.71, 95% CI (0.32, 1.09)] (Figure 3).

**Figure 8:**
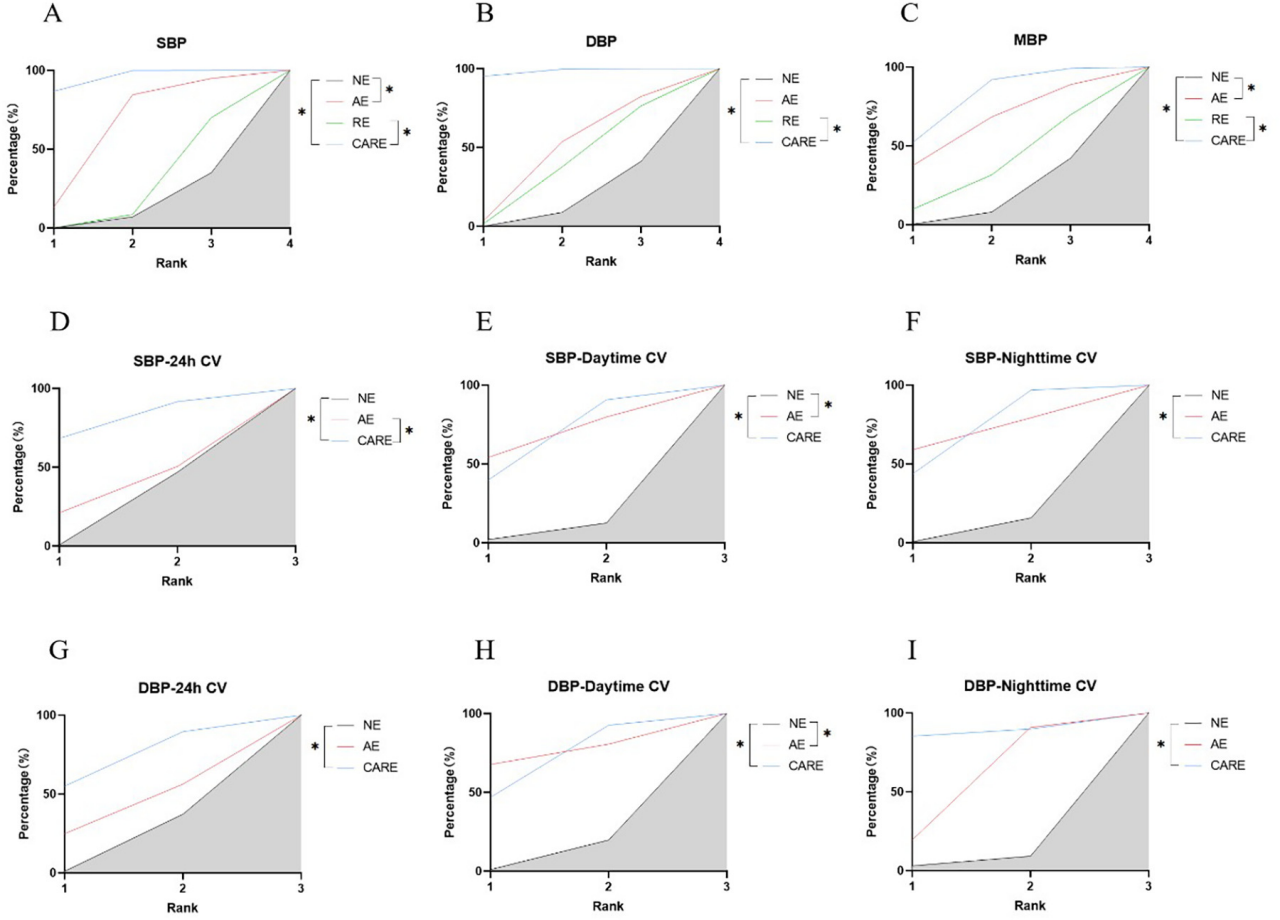
SUCRA plots of the effects of aerobic exercise, resistance exercise, and combined aerobic resistance exercise on the blood pressure and blood pressure variability of patients with hypertension. **(A)** Systolic blood pressure (SBP); **(B)** diastolic blood pressure (DBP); **(C)** mean blood pressure (MBP); **(D)** 24-hour systolic blood pressure coefficient of variation (SBP-24h CV); **(E)** daytime systolic blood pressure coefficient of variation (SBP-Daytime CV); **(F)** nighttime systolic blood pressure coefficient of variation (SBP-Nighttime CV); **(G)** 24-hour diastolic blood pressure coefficient of variation (DBP-24h CV); **(H)** daytime diastolic blood pressure coefficient of variation (DBP-Daytime CV); **(I)** nighttime diastolic blood pressure coefficient of variation (DBP-Nighttime CV); *p < 0.05. Note: SUCRA, Surface under the cumulative ranking curve; The literature on resistance training lacks data on blood pressure variability indicators; Therefore, only a network analysis was conducted on the effects of aerobic exercise and combined aerobic resistance exercise on this indicator; NE, no exercise; AE, aerobic exercise; RE, resistance exercise; CARE, Combined aerobic resistance exercise.

**Figure 9:**
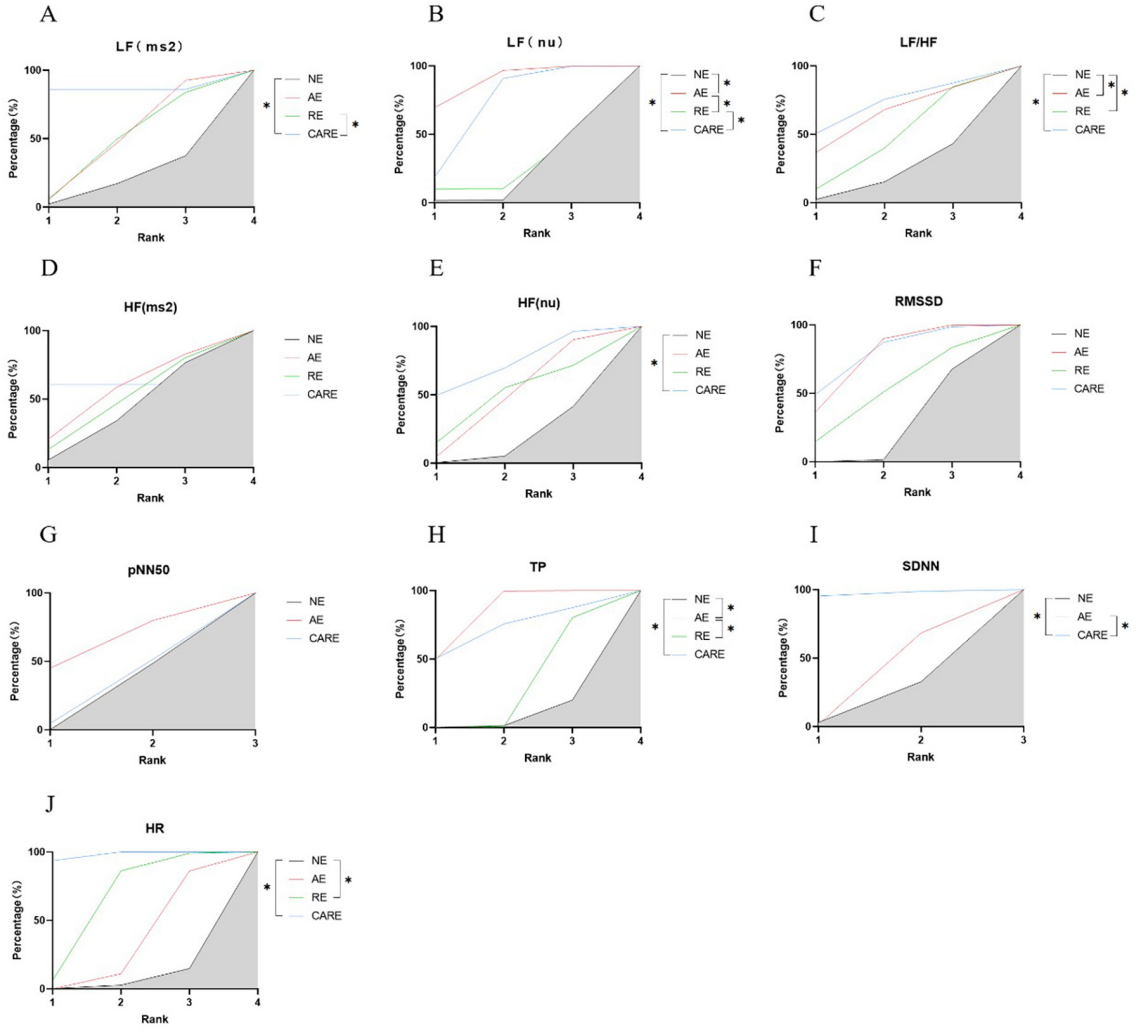
SUCRA plots of the effects of aerobic exercise, resistance exercise, and combined aerobic and resistance exercise on the heart rate variability in patients with hypertension. **(A)** low frequency power (LF); **(B)** Normalized low-frequency power; **(C)** low frequency power to high frequency power ratio (LF/HF); **(D)** high frequency power (HF); **(E)** Normalized high-frequency power; **(F)** root mean square of successive RR interval differences (RMSSD); **(G)** the number of successive RR interval differences greater than 50ms accounted for the total RR Percentage of the number of phases (pNN50); **(H)** total power (TP); **(I)** standard deviation of all normal sinus RR intervals (SDNN); **(J)** heart rate (HR); *p < 0.05. Note: The literature on resistance training lacks data on pNN50 and SDNN indicators; Therefore, only network analyses were conducted on the effects of aerobic exercise and combined aerobic resistance exercise on these indicators.

Aerobic and combined aerobic resistance exercise significantly reduced SBP in hypertensive patients (*p* < 0.05). Aerobic combined resistance exercise had a significantly higher effect on SBP than resistance exercise (*p* < 0.05) (Figure 8A). Aerobic combined resistance exercise significantly reduced DBP in hypertensive patients (*p* < 0.05). Aerobic combined resistance exercise had a significantly higher effect on DBP than resistance exercise (*p* < 0.05) (Figure 8B). Aerobic combined resistance exercise significantly reduced MBP in hypertensive patients (*p* < 0.05). Aerobic combined resistance exercise had a significantly higher effect on MBP than resistance exercise (*p* < 0.05) (Figure 8C). Resistance and aerobic combined resistance exercise significantly reduced HR in hypertensive patients (*p* < 0.05) (Figure 9J).

#### 3.2.2 Results of the effect of exercise on blood pressure variability in hypertensive patients

Exercise significantly reduced the joint effect size of hypertension being a patient's blood pressure variability [WMD = 0.89, 95% CI (0.51, 1.27)]. The exercise did not show significant reduction in SBP-24 h CV [WMD = 1.15, 95% CI (−0.48, 2.78)], SBP-Daytime CV [WMD = 0.71, 95% CI (−0.00, 1.42)], and SBP-Nighttime CV [WMD = −0.05, 95% CI (−1.88, 1.77)]. The exercise significantly reduced the DBP-24 h CV [WMD = 1.85, 95% CI (0.38, 3.31)], DBP-Daytime CV [WMD = 1.46, 95% CI (0.98, 1.94)] and DBP-Nighttime CV [WMD = 0.48, 95% CI (0.10, 0.85)] (Figure 4).

Aerobic combined resistance exercise significantly reduced SBP-24 h CV in hypertensive patients (*p* < 0.05). Aerobic combined resistance exercise had a significantly higher effect on SBP-24 h CV than aerobic exercise (*p* < 0.05) (Figure 8D). Aerobic and aerobic combined resistance exercise significantly reduced SBP-Daytime CV in hypertensive patients (*p* < 0.05) (Figure 8E). Aerobic combined resistance exercise significantly reduced SBP-Nighttime CV in hypertensive patients (*p* < 0.05) (Figure 8F). Aerobic combined resistance exercise significantly reduced DBP-24 h CV in hypertensive patients (*p* < 0.05) (Figure 8G). Aerobic and combined aerobic resistance exercise significantly reduced DBP-Daytime CV in hypertensive patients (*p* < 0.05) (Figure 8H). Aerobic and combined aerobic resistance exercise significantly reduced DBP-Nighttime CV in hypertensive patients (*p* < 0.05) (Figure 8I).

#### 3.2.3 Results of the effect of exercise on the parasympathetic nervous system in hypertensive patients

The effect of exercise on the joint effect size of the parasympathetic nervous system in hypertensive patients was not significant [SMD = −0.08, 95% CI (−0.31, 0.14)]. Exercise did not significantly increase HF (ms²) [SMD = −0.09, 95%CI (−0.70, 0.52)], HF(nu) [SMD = −0.14, 95%CI (−0.46, 0.17)], RMSSD [SMD = −0.04, 95%CI (−0.48, 0.41)], and pNN50 [SMD = −0.07, 95%CI (−0.50, 0.36)] in patients with hypertension (Figure 5).

Aerobic combined resistance exercise significantly increased HF (nu) in hypertensive patients (*p* < 0.05) (Figure 9E).

#### 3.2.4 Results of the effect of exercise on the balance of the sympathetic nervous system as well as the autonomic nervous system in patients with hypertension

Exercise significantly reduced the joint effect sizes of sympathetic as well as autonomic nervous system homeostasis in hypertensive patients [SMD = 0.29, 95% CI (0.17, 0.40)]. Exercise had a significant reduction effect on LF (ms²) [SMD = 0.23, 95%CI (0.03, 0.42)], LF (nu) [SMD = 0.28, 95%CI (0.08, 0.48)], and LF/HF [SMD = 0.37, 95%CI (0.16, 0.58)] in hypertensive patients. Combined with the results of the effects of exercise on the parasympathetic nervous system in hypertensive patients, it suggested that exercise improves the balance of the autonomic nervous system in hypertensive patients mainly focusing on the sympathetic nervous system (Figure 6).

Aerobic combined resistance exercise significantly reduced LF (ms²) in hypertensive patients (*p* < 0.05). Aerobic combined resistance exercise had a significantly higher effect on LF (ms²) than resistance exercise (*p* < 0.05) (Figure 9A). Aerobic and aerobic combined resistance exercise significantly reduced LF (nu) in hypertensive patients (*p* < 0.05). Aerobic combined resistance exercise had a significantly higher effect on LF (nu) than resistance exercise (*p* < 0.05) (Figure 9B). Aerobic, resistance and aerobic combined resistance exercise significantly reduced LF/HF in hypertensive patients (*p* < 0.05) (Figure 9C).

#### 3.2.5 Results of the effect of exercise on the combined frequency-domain and time-domain effects of heart rate variability in hypertensive patients

Exercise significantly increased the joint effect size of the combined frequency-domain and time-domain effects of heart rate variability in hypertensive patients [SMD = −0.85, 95% CI (−1.30, −0.40)]. Exercise had a significant increase on TP [SMD = −0.94, 95% CI (−1.61, −0.26)] and SDNN [SMD = −0.70, 95% CI (−1.30, −0.11)] in hypertensive patients (Figure 7).

Aerobic and aerobic combined resistance exercise significantly increased TP in hypertensive patients (*p* < 0.05). The effect of aerobic exercise on TP was significantly higher than resistance exercise (*p* < 0.05) (Figure 9H). Aerobic combined resistance exercise significantly increased SDNN in hypertensive patients (*p* < 0.05). Aerobic combined resistance exercise had a significantly higher effect on TP than aerobic exercise (*p* < 0.05) (Figure 9I).

## 4 Discussion

This study analyzed the effect of exercise on ANS in hypertensive patients with the intention of determining the efficacy of exercise on SNS as well as PNS specifically in hypertensive patients. And the effects of specific forms of exercise including aerobic, resistance and aerobic combined resistance exercise on ANS in hypertensive patients were comparatively analyzed to explore more suitable forms of exercise for hypertensive patients to practice. This study found that either aerobic, resistance, or aerobic combined with resistance exercise can have beneficial effects (decrease in systolic and diastolic blood pressure, improvement in heart rate) in hypertensive patients.

### 4.1 Effect of exercise on blood pressure variability in hypertensive patients

Blood pressure variability (BPV) refers to fluctuations in blood pressure over time, and there is growing evidence that BPV is an independent predictor of cardiovascular risk (21). Any means of inducing a reduction in blood pressure variability may be a complementary therapeutic target for blood pressure control in hypertensive patients (22). Physical activity as a nonpharmacologic strategy for the prevention and treatment of hypertension (23). In turn, traditional exercises such as aerobic and resistance training are the focus of these studies (24). A recent meta-analysis suggests that aerobic exercise is a cost-effective way to improve BPV and demonstrates that aerobic exercise reduces BPV, in adult hypertensive patients (25). In this study, this phenomenon was also observed, especially in terms of the improvement of diastolic blood pressure. In addition, BPV has an important association with the autonomic cardiovascular system, and higher BP fluctuations may be associated with damage to the ANS, leading to hypertension (26). For example, in a meta-analysis exploring the effects of resistance training on autonomic control of the heart in healthy and diseased individuals, it was found that resistance training had little to no effect on autonomic control of the heart in healthy individuals, but it improved autonomic control of the heart in patients with (27). Although the mechanisms associated with the modulation of BPV after exercise are not fully understood, the reduction in blood pressure variability appears to be achieved by decreasing sympathetic activity and systemic vascular resistance (28).

### 4.2 Effects of exercise on the sympathetic and parasympathetic nervous system in hypertensive patients

The cardiovascular response to exercise is largely determined by neurocirculatory control mechanisms, which help regulate vascular resistance and, together with local vasodilatory mechanisms, promote blood flow to active muscles and organs (29). In a meta-analysis focused on exploring the effects of exercise training on muscle sympathetic nerve activity (MSNA), researchers found that individuals with cardiovascular disease had more significant changes in MSNA compared to individuals without cardiovascular disease. This analysis revealed an association between exercise and response, as evidenced by the fact that individuals whose sympathetic nerve activity was at a higher level prior to the exercise intervention showed a more significant decrease after receiving the exercise intervention. This finding may be valuable in improving cardiovascular health (30). Exercise has been shown to reduce elevated SNS activity in heart failure, hypertension, and the aging heart and vascular system (31). For example, 4 months of aerobic training reduced MSNA and enhanced arterial pressure reflex regulation in patients with hypertension (32). In contrast, activation of cardiac sympathetic modulation was also found in a meta-analysis of healthy individuals exposed to acute resistance exercise (33). In addition, it has been shown that the sympathetic drive reduction after exercise is more pronounced in patients with essential hypertension than in normotensive populations, which may underlie the antihypertensive effects of exercise (11). During recovery from exercise, vasodilation and reduced sympathetic nerve activity contribute significantly to post-exercise hypotension (34). These studies provide some evidence that exercise-induced blood pressure reductions in hypertensive patients may be dominated by sympathetic mechanisms.

In addition, the second arm of the ANS is the PNS, which regulates a wide range of functions from blood pressure and heart rate to respiration and immune responses (35). Reactivation of the PNS can be measured by heart rate recovery (reduction in heart rate after exercise). In the elderly, physical activity also has important antiarrhythmic effects and improves the influence of vagal components on hemodynamic parameters (36). Long-term aerobic exercise significantly reduces sympathetic excitability and improves sympathetic and vagal balance, leading to quiet heart rate and blood pressure reduction (37). Furthermore, in a recent meta-analysis the benefits of aerobic combined with resistance exercise in improving the vagus nerve in middle-aged hypertensive women were found, yet two other systematic studies negated the role of resistance exercise in optimizing vagal regulation (7, 15, 38, 39). Suggesting the controversy of exercise for parasympathetic nervous system improvement. In addition, it has been shown that when arterial pressure falls, the SNS is immediately activated, leading to increased cardiac output and peripheral vasoconstriction (40). In contrast to increased sympathetic activity, parasympathetic activity is decreased in hypertensive patients, indicating an imbalance between sympathetic and parasympathetic activity (41). However, the specific effect of exercise on SNS as well as PNS in hypertensive patients remains unclear.

Heart rate variability (HRV) not only expresses ANS status, but also assesses PNS and SNS activity (42, 43). A growing number of studies have linked autonomic nervous system imbalances (assessed by HRV) to several pathophysiologic conditions, particularly in cardiovascular disease (44). For example, 12 weeks of aerobic combined resistance training significantly increased HF (nu) and decreased the LF/HF ratio in diabetics (38). In another study, 4 weeks of aerobic exercise training (20 min at 80% of maximum heart rate intensity) improved SDNN and total power and reduced LF, LF/HF, and systolic and diastolic blood pressure in patients with prehypertension (15). Similarly, 4 weeks of resistance exercise effectively lowered systolic blood pressure and slightly improved HRV frequency domain index in hypertensive patients, thereby modulating cardiac autonomic balance (7, 45). In the present study, we also used HRV (time and frequency domains) to analyze the effect of exercise on SNS as well as PNS specifically in hypertensive patients. In subgroup analyses targeting the frequency domain of HRV, statistically significant results were found for LF (ms²), LF/HF, and TP, suggesting improved ANS function. However, further analysis of normalized LF (nu) and HF (nu) revealed that HF, which responds to fluctuations in PNS, did not change significantly in response to the intervention of movement, whereas LF was mainly driven by SNS, and this decrease suggests a negative transfer of SNS activity (46). However, there is still some controversy regarding the interpretation of LF, and it has been suggested that LF (which is co-regulated by the SNS and PNS) may reflect sympathetic-parasympathetic balance rather than merely sympathetic activity (47). Thus, LF cannot be used alone as the sole indicator of sympathetic activity, but needs to be combined with other parameters. The RMSSD and pNN50, which are closely related to the PNS, were also found not to be significantly altered by the intervention of exercise in a subgroup analysis targeting the HRV time domain (13), and SDNN showed a significant increase, which likewise proves the major modulating role of the SNS in the improvement of the symptoms of patients with hypertension by exercise.

### 4.3 Effects of aerobic, resistance, and combined exercise on the autonomic nervous system in hypertensive patients

The form of exercise has a great influence on the improvement of the functioning of the organism. For example, exercise training, especially aerobic exercise, is a well-recognized tool for lowering blood pressure in hypertensive subjects and for avoiding elevated blood pressure in prehypertension (6). Resistance exercise has also been proposed for the control of hypertension, but it has been reported to have a smaller antihypertensive effect compared to aerobic exercise (48). However, one study found that a combined exercise approach was more acceptable to hypertensive patients and had higher long-term adherence in an unsupervised home training program (49). Over time, this may translate into a more sustained blood pressure response. In addition, the combination of aerobic and resistance exercise can elicit additional positive adaptations in the cardiovascular system, which, in addition to the effects on blood pressure, may help reduce overall cardiovascular risk in hypertensive subjects (50). Similarly, the recommendations of the American College of Sports Medicine (ACSM) specify aerobic and resistance exercise in programs for people with hypertension, and combined exercise of the two showed more significant improvements in blood pressure than exercise alone (51). Although the role of aerobic and resistance training in enhancing cardiovascular fitness is well documented in the scientific literature, the efficacy of its impact on ANS control remains unclear (38). Based on the existing literature, we conducted a retrospective analysis of the effects of aerobic exercise, resistance exercise and combined exercise on blood pressure regulation and ANS in hypertensive patients. The results showed that aerobic exercise combined with resistance exercise has the best effect on ANS (especially SNS), which suggests that in the future, the combination of the two should be emphasized in the treatment of hypertension, and the time and intensity of the combination should be constantly improved and updated.

### 4.4 Limitations

Only published literature was included in this study, excluding unpublished literature, which may have affected the comprehensiveness of this study. The included literature did not discuss the form of medication (type of medication, duration of medication use), which may have introduced bias due to differences in medication use. In addition, in the literature examining resistance exercise on the autonomic nervous system in hypertensive patients, there are fewer outcome indicators involving blood pressure variability, which may have influenced the judgment of resistance exercise in this study. Although the included studies demonstrated rigor in randomization, uncertainty remains regarding blinding and transparency of reporting. Therefore, the credibility of their conclusions needs to be assessed with caution (52). Egger's test was used to assess publication bias, and the results of MBP as well as blood pressure variability suggested the presence of potential publication bias, which may be influenced by the small sample effect. The limited number of studies may lead to insufficient statistical power, which may affect the interpretation and reliability of the results (53). Heterogeneity (*I*² ≥ 50%) was present among the included studies. It may mainly stem from the fact that: the age range of hypertensive patients in each study ranged from 38 ± 5 to 68 ± 7 years, with different autonomic nervous system functions and responses to exercise in patients of different ages; and the duration of hypertension affects the degree of autonomic nervous system damage and adaptability to exercise interventions (54). In addition, because the included literature did not differentiate between genders, hypertensive patients of different genders may have differentiated autonomic nervous system modulation patterns and exercise adaptations due to differences in hormone levels (55). In addition, the details of the exercise protocols varied, such as the intensity of exercise from 50% to 75% of maximal heart rate for aerobic exercise and the percentage of individual maximal repetitions for resistance exercise, and these differences may have increased the uncertainty of the study results and reduced the stability of the combined effect sizes (56). To further identify potential sources of heterogeneity, this study conducted a sensitivity analysis. This analysis considered all outcome indicators and systematically excluded each study to see its effect on the overall joint effect size. The results suggest that the impact of individual studies on the overall analyzed results is limited. However, despite the results of this meta-analysis, limitations still exist. Furthermore, as the gold standard for measuring sympathetic nerve activity, muscle sympathetic nerve activity (MSNA) was not included in the analysis due to the low availability of relevant literature and the insufficient compatibility with the non-invasive indicators of this study (57). This may have limited the depth of assessment of the autonomic nervous system response. Future studies need to be more rigorous in the design, implementation, and analysis phases to obtain more accurate and generalizable conclusions to provide a more reliable basis for exercise interventions in hypertensive patients.

## 5 Conclusion

Exercise can regulate hypertensive patients through the autonomic nervous system, and can significantly improve the clinical symptoms of hypertensive patients. Moreover, the autonomic nervous system regulation of hypertensive patients by exercise mainly focuses on sympathetic nerves. Aerobic, resistance, and a combination of both exercise modalities all have positive effects on hypertensive patients. Among them, the efficacy of combined exercise in affecting the autonomic nervous system (especially the sympathetic nervous system) of hypertensive patients is more obvious, and it is more effective in improving the blood pressure and heart rate level of the patients. These results provide valuable guidance for clinical complementary therapies, suggesting that future investigations into the mechanisms of exercise interventions in hypertensive patients could focus on the sympathetic nervous system, and that the protocol for exercise to improve hypertension could focus on aerobic combination training, with continual refinement and updating of the duration as well as the intensity of this form of exercise.

## Data Availability

The original contributions presented in the study are included in the article/Supplementary Material, further inquiries can be directed to the corresponding author.
